# Fibrosis: a structural modulator of sinoatrial node physiology and dysfunction

**DOI:** 10.3389/fphys.2015.00037

**Published:** 2015-02-12

**Authors:** Thomas A. Csepe, Anuradha Kalyanasundaram, Brian J. Hansen, Jichao Zhao, Vadim V. Fedorov

**Affiliations:** ^1^Department of Physiology and Cell Biology, Davis Heart and Lung Research Institute, The Ohio State University Wexner Medical CenterColumbus, OH, USA; ^2^Auckland Bioengineering Institute, The University of AucklandAuckland, New Zealand

**Keywords:** sinoatrial node, fibrosis, sinus node dysfunction, aging, exit block, sinoatrial reentry, atrial fibrillation, heart failure

## Abstract

Heart rhythm is initialized and controlled by the Sinoatrial Node (SAN), the primary pacemaker of the heart. The SAN is a heterogeneous multi-compartment structure characterized by clusters of specialized cardiomyocytes enmeshed within strands of connective tissue or fibrosis. Intranodal fibrosis is emerging as an important modulator of structural and functional integrity of the SAN pacemaker complex. In adult human hearts, fatty tissue and fibrosis insulate the SAN from the hyperpolarizing effect of the surrounding atria while electrical communication between the SAN and right atrium is restricted to discrete SAN conduction pathways. The amount of fibrosis within the SAN is inversely correlated with heart rate, while age and heart size are positively correlated with fibrosis. Pathological upregulation of fibrosis within the SAN may lead to tachycardia-bradycardia arrhythmias and cardiac arrest, possibly due to SAN reentry and exit block, and is associated with atrial fibrillation, ventricular arrhythmias, heart failure and myocardial infarction. In this review, we will discuss current literature on the role of fibrosis in normal SAN structure and function, as well as the causes and consequences of SAN fibrosis upregulation in disease conditions.

## Introduction

“*We might mention also that, in some of the pathological hearts cut by us, sections of this region* (i.e., the sinoatrial node) *appeared to show a definite increase in the amount of fibrous tissue present-a fact of considerable importance…*”

(Keith and Flack, 1907)

In the human heart, cardiac rhythm is initiated and regulated by the primary pacemaker of the heart, the Sinoatrial Node (SAN) (Keith and Flack, 1907; Lewis et al., 1910; James, 1961; Boineau et al., 1988; Opthof, 1988; Boyett et al., 2000; Chandler et al., 2009; Fedorov et al., 2010b). Initiation of heart rhythm occurs within specialized cardiomyocytes of the SAN and is propagated throughout the atria and ventricles by the cardiac conduction system. Sinus Node Dysfunction (SND), also referred to as Sick Sinus Syndrome (SSS), commonly translates into rhythm abnormalities manifested as brady-arrhythmias or tachycardia-bradycardia (tachy-brady) syndrome (Mangrum and DiMarco, 2000), which are frequently associated with cardiac diseases including atrial fibrillation (AF), malignant ventricular arrhythmias, heart failure (HF) and cardiac arrest (Luu et al., 1989; Sumitomo et al., 2007; Faggioni et al., 2013; Hjortshoj et al., 2013; Alonso et al., 2014; Jensen et al., 2014). With the aging population, it is projected that the annual incidence of SND cases in the US will increase from 78,000 in 2012 to 172,000 in 2060 (Jensen et al., 2014). SND is the predominant prognosis for electronic pacemaker implantation (Mangrum and DiMarco, 2000; Packer et al., 2009; Greenspon et al., 2012), emphasizing the important role that the SAN plays in maintaining normal cardiac rhythm and in human arrhythmic diseases.

Since the discovery of the SAN by Keith and Flack in 1907, significant strides in our understanding of SAN pacemaker function (Lakatta and DiFrancesco, 2009) have allowed for new and exciting therapeutic strategies to treat SAN disease, such as the development of Ivabradine as a selective drug against inappropriate SAN tachycardia (Cappato et al., 2012) and artificial biological pacemakers (Miake et al., 2002; Rosen et al., 2004; Rosen, 2014). The heterogeneous distribution of specialized ion channels, intracellular sodium/calcium handling proteins, gap junction channels and receptors within the SAN pacemaker complex are a few of the critical players shown to be involved in SAN pacemaking that have been addressed in recent reviews (Monfredi et al., 2010; Dobrzynski et al., 2013; Wu and Anderson, 2014). In addition to these molecular mechanisms, the passive, structural features of the SAN complex also contributes significantly to its normal functioning.

In contrast to the simplified SAN structure in many textbooks, studies in both human and canine hearts have revealed that the SAN is a complex multi-compartment structure (James, 1961; Opthof, 1988; Boineau et al., 1989; Beau et al., 1995; Boyett et al., 2000; Sanchez-Quintana et al., 2005; Chandler et al., 2009; Fedorov et al., 2009, 2010a). The SAN, in almost all mammalian hearts, is characterized by clusters of specialized cardiomyocytes, enmeshed within strands of connective tissue or fibrosis, mostly a combination of collagen, elastin and fibroblasts (Lev, 1954; Hudson, 1960; Truex et al., 1967; Sanchez-Quintana et al., 2002). This fibrotic matrix provides mechanical protection (Alings et al., 1995) of the SAN and electrically insulates the SAN pacemaker cells from the surrounding atrial myocardium, thereby efficiently regulating normal sinus rhythm. This review will take a more in depth look at the role of fibrosis in normal SAN function, as well as factors involved in unfavorable fibrosis production observed in patients and animal models with cardiac diseases and SND (Liu et al., 2007; de Jong et al., 2011; Nakao et al., 2012; Glukhov et al., 2013, 2015; Alonso et al., 2014; Jensen et al., 2014; Morris and Kalman, 2014).

## Fibrosis in normal SAN function

### Fibrosis defines SAN structure and maintains functional integrity of the pacemaker complex

**Figure 1:**
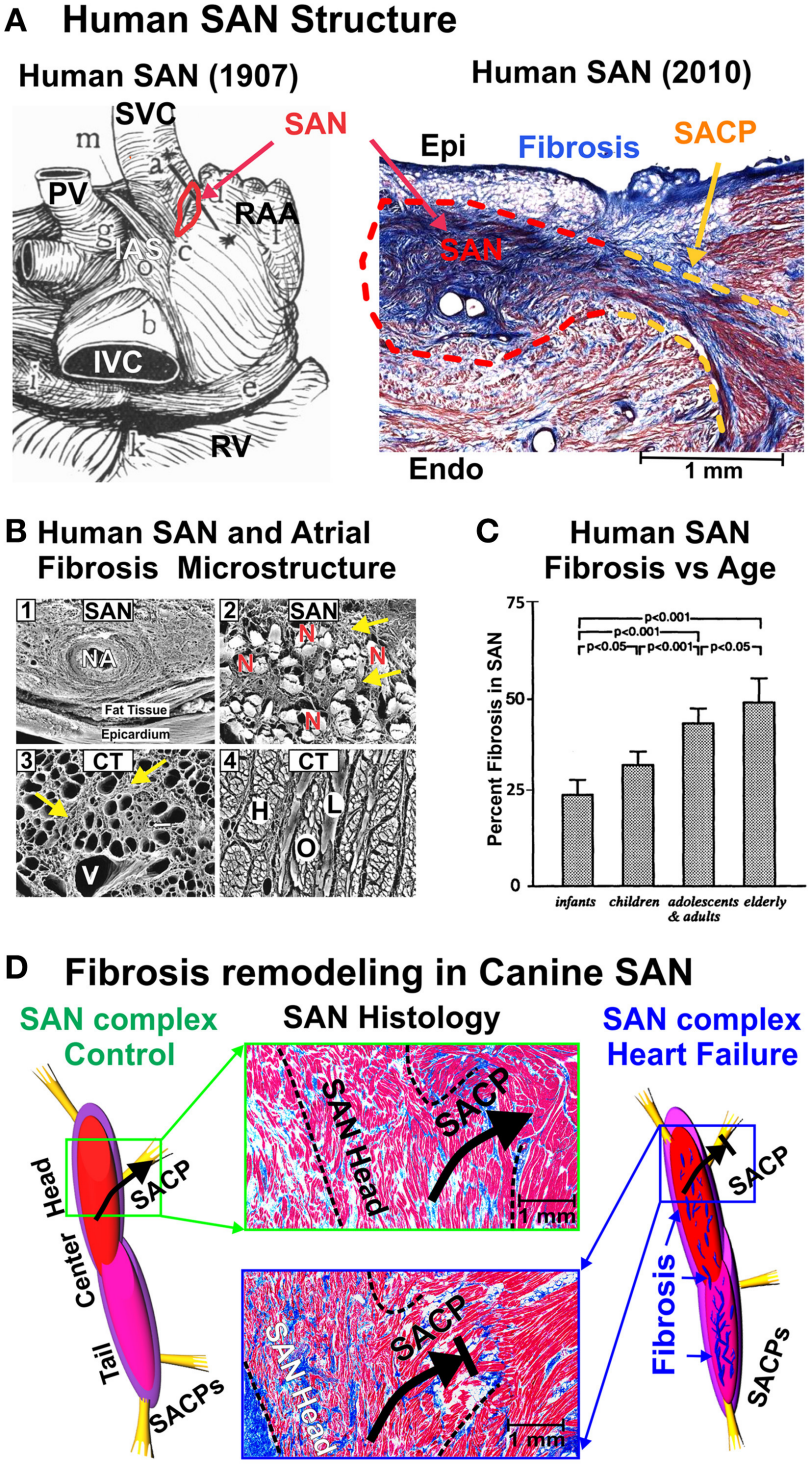
**(A)** Left: Drawing of the posterior human atrial anatomy from (Keith and Flack, 1907), with SAN outlined in red. Right: Histological cross section of the SAN (red outline) connected to the atria by a SACP (yellow outline). The SAN is seen to be isolated from the atria by sup-epicardial fat and connective tissue. Modified from Fedorov et al. (2010b, 2012) with permission. **(B)** 1—Scanning electron micrograph of the SAN and nodal artery (NA) of a cross section through the CT after digestion of the nodal cells. The collagenous sheaths of endomysial fibrosis formed a complex network in the gaps between the nodal cells, whereas occasional perimysial septums were seen between them. 2—Scanning electron micrograph of non-macerated SAN cells (N), which are encased in dense and coarse endomysial sheaths (yellow arrows) in a specimen from a 75 year old. 3—Scanning electron micrograph of a cross section through the CT, after digestion, from a specimen of 70 years shows a diffuse notable excess of endomysial sheaths (yellow arrows) indicating focal interstitial reactive fibrosis. Vascular space (V) corresponds to a coronary vein. 4—Scanning electron micrograph of non-macerated cross section through the body of the CT shows mainly longitudinal fibers (H) with intermingling oblique (O) or lateral (L) fibers. From Sanchez-Quintana et al. (2002) with permission. **(C)** Graph showing percentage of fibrous connective tissue volume to the total SAN volume, mean value, and standard deviation. From Shiraishi et al. (1992); used with permission. **(D)** Fibrosis in the SAN is increased in HF vs. control dog. Left: A structural model of the control canine SAN complex and SACPs. Middle: histological sections showing upregulated fibrotic and fatty content in SAN complex (black outline) and SACP in HF (bottom, blue box) compared to control (top, green box). Right: A structural model of the heart failure SAN complex and SACPs, showing upregulated intranodal fibrosis (blue fibrotic strands). Modified from Lou et al. (2014) with permission. Abbreviations: CT, crista terminalis; Endo, endocardium; Epi, epicardium; HF, heart failure; IAS, interatrial septum; PV, pulmonary veins; RAA, right atrial appendage; RV, right ventricle; SACP, sinoatrial conduction pathway; SAN, sinoatrial node; SVC and IVC, superior and inferior vena cava.

The SAN is anatomically located at the junction of the superior vena cava and right atrium in the mammalian heart (Figure 1A). In the normal adult human heart, the SAN is 12–20 mm long and 2–6 mm wide, identified by its ellipsoidal shape that traverses intramurally. The superior part (head) lies about 1 mm beneath the epicardium, separated by a layer of connective tissue and fat (Keith and Flack, 1907; James, 1961; Truex et al., 1967; Matsuyama et al., 2004; Sanchez-Quintana et al., 2005). The SAN spreads from its head inferiorly for 10–20 mm remaining beneath the sulcus terminalis and just above the crista terminalis and has several extensions into the surrounding atrial myocardium, forming the specialized sinoatrial conduction pathways (SACPs) (Lev, 1954; Hudson, 1960; Demoulin and Kulbertus, 1978; Fedorov et al., 2010b) (Figure 1A). Importantly, the SAN consists of small clusters of pacemaker myocytes, arranged in parallel rows that frequently anastomose. Dense connective tissue, nerve fibers, and capillaries are interspersed with the SAN pacemaker clusters, creating the SAN pacemaker complex.

A century ago, Keith and Flack's pioneering studies described fibrotic insulation in the human SAN: “In the human heart the fibers (i.e., SAN pacemaker cell clusters) are striated, fusiform, with well-marked elongated nuclei, plexiform in arrangement, and embedded in *densely packed connective tissue*” (Keith and Flack, 1907). Since then, extensive investigations of the human SAN structure by many others (Lev, 1954; Hudson, 1960; James, 1961; Truex et al., 1967; Demoulin and Kulbertus, 1978; Matsuyama et al., 2004) have confirmed the extensive fibrotic pattern within the node. Fibrosis provides an encompassing, honeycomb-like, structural support composed of a network of collagen sheaths of endomysium (Sanchez-Quintana et al., 2002) (Figure 1B). These data show that interstitial fibrosis within the normal SAN builds scaffolding to “house” the sophisticated SAN myocytes and may be necessary to maximize electrical insulation and safe propagation in the SAN complex (Davies and Pomerance, 1972; Fedorov et al., 2010b, 2012) (Figure 1B). Furthermore, the human SAN is electrically insulated from the surrounding atria by fibrosis and fatty tissue (Lev, 1954; Sanchez-Quintana et al., 2005; Monfredi et al., 2010; Fedorov et al., 2010b) except for distinct SACPs, which electrically connect the SAN to the right atrium (Fedorov et al., 2012). Integral to SAN function, fibrotic and fatty insulation of the SAN prevents the depression of pacemaker automaticity from the hyperpolarizing electrical load of the atrial myocardium (Joyner and van Capelle, 1986).

### SAN fibrosis increases with heart size and age

Considerable physiological and structural evidence support the generalization that large mammals (including human, dog, pig, yak, horse, etc.) have a more compact and distinct SAN structure than smaller mammals (Keith and Flack, 1907; James, 1961; Opthof et al., 1987; Fedorov et al., 2009, 2012; Duan et al., 2012; Lou et al., 2013, 2014; Glukhov et al., 2015). Histological studies clearly demonstrated that interstitial fibrosis is an inherent component of normal SAN structure and can be traced from newborn hearts (Lev, 1954; Shiraishi et al., 1992; Alings et al., 1995). The size of the heart, as well as its mechanical demands, increases dramatically throughout life and may have a major effect on SAN structure and function. In 1954, Lev published the first detailed study of structural changes of the SAN with age in 54 human SAN samples ranging from 4 months gestation to 90 years of age; this exhaustive investigation revealed that the SAN tissue was already clearly discernable from the atrial tissue at 5 months gestation, particularly due to the greater abundance of fibroblasts and distinctly higher number of collagen fibers (Lev, 1954). Collagen content in the human infant SAN is about 24% and this number climbs to about 70% fibrosis in the adult heart (Shiraishi et al., 1992; Alings et al., 1995) (Figure 1C). This natural correlation between aging and increased fibrotic content in the SAN has also been documented in animal models, for example SAN fibrosis in mouse rises from 12–17% at 3 months to 23–25% at 12 months (Hao et al., 2011; Glukhov et al., 2015). The age-induced increase of SAN fibrosis is strongly correlated with slowed intrinsic heart rate as well as slowed SAN conduction in human and mammalian hearts (Kuga et al., 1993; Sanders et al., 1994; Noujaim et al., 2004; Hao et al., 2011; Akoum et al., 2012). The SAN modeling study by Oren and Clancy (2010) is in agreement with a study by Fahrenbach et al. (2007): fibrosis correlates with slowed intrinsic conduction through two mechanisms: (1) heterocellular coupling enables the fibroblast to depolarize the cardiomyocytes (or act as a current sink), (2) fibroblasts physically separate the pacemaker cardiomyocytes, slowing SAN rhythm through a reduction in mutual entrainment (Jalife, 1984).

Apart from aging, a natural size-to-fibrotic content relationship is evidenced in the gradual increase in SAN percent fibrosis as the size of the species increases. The adult mouse SAN is composed of less fibrosis (Hao et al., 2011; Glukhov et al., 2015) than the adult cat SAN, which consists of 27% fibrosis (Alings et al., 1995). The healthy adult canine SAN has a range of 22–30% fibrosis (Glukhov et al., 2013; Lou et al., 2014), while the normal adult human SAN shows roughly 45% fibrosis (Alings et al., 1995). The contribution of increased intranodal fibrosis in large mammals to their functional demands is not completely clear, but as the atrial tissue increases in large vs. small hearts, more insulation around the SAN may be necessary to protect it from the electrical and mechanical load of the atria (Joyner and van Capelle, 1986). Universally, large mammals have slower heart rates vs. smaller animals as established by Noujaim et al.'s multi-species study (Noujaim et al., 2004). Based on the studies discussed above, we suggest that intranodal fibrosis is inversely correlated with intrinsic SAN rhythm and conduction, and directly correlated with age and heart size.

### Fibrosis provides mechanical protection for the SAN and prevents pathophysiological heart rate responses to atrial stretch and pressure

In addition to structural scaffolding and electrical insulation from the atria, interstitial fibrosis within the SAN can prevent it from increased interstitial volume changes due to stretch or compression. As the natural growth during the life span or size of the animal increases, the size and thickness of the atria also increases to meet contractile demands (Sanchez-Quintana et al., 2002). Mechanical stretch is a powerful myogenic modulator of SAN function (Brooks and Lange, 1977); in fact, possibly as a local regulator of heart rate, stretch and/or increased intra-atrial pressure can increase SAN rhythm, whereas compression and/or increased arterial pressure in the SAN artery may lead to slowing of SAN rhythm (James and Nadeau, 1962; Lange et al., 1966; Hashimoto et al., 1967). These local responses to stretch and pressure may be carried out through stretch-activated channels in pacemaker cells and SAN fibroblasts, which have been shown to electrically couple with SAN myocytes in some studies (Kohl et al., 1994; Kamkin et al., 2003; Cooper and Kohl, 2005; Fahrenbach et al., 2007; Oren and Clancy, 2010). By providing a relatively tough casing for the compact SAN, fibrosis could prevent the node from overstretching due to the mechanical pressures of the atrial myocardium (Alings et al., 1995).

## Fibrosis in SAN dysfunction

### Increasing fibrosis within the SAN pacemaker complex is associated with SND and arrhythmias

**Figure 2:**
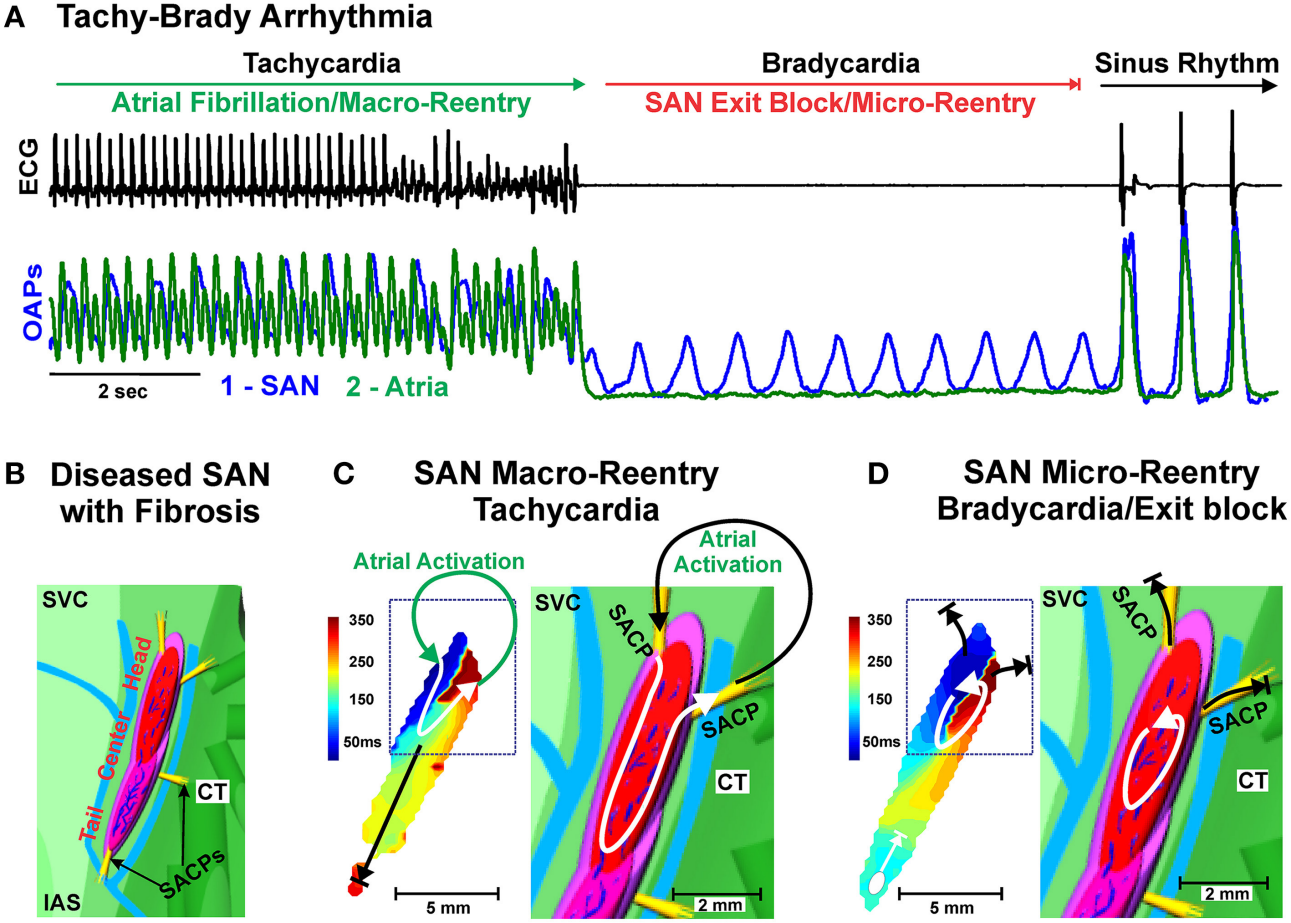
**(A)** Example of Tachy-Brady arrhythmia often observed in structurally remodeled hearts with upregulated intranodal fibrosis. Atrial Fibrillation or Tachycardia leads to Bradycardia (long pauses) due to SAN exit block, followed by recovery of sinus rhythm or new Tachycardia event. ECG (black) and Optical Action Potentials (green and blue). Modified from Lou et al. (2013) with permission. **(B)** Enlarged epicardial view of 3D canine SAN model based on structural and functional data from optical mapping experiments. The SAN is demarcated from the atrium (green) by 3 bifurcating coronary arteries (light blue) and connective tissue (light purple). The yellow bundles show SACPs that electrically connect the SAN to the atrium. **(C)** Macro-reentry between the SAN and atria that occurred in the structurally remodeled canine heart after cessation of atrial tachypacing. Path of macro-reentry correlated with fibrotic strands within SAN. **(D)** SAN micro-reentry transformed from the previous macro-reentry is shown. Fibrosis strands were the structural substrates anchoring micro-reentry to this area. (**B–D)** modified from Glukhov et al. (2013) with permission. Abbreviations as seen in Figure 1.

SND is conceptualized as a spectrum of heart rhythm disturbances including bradycardia, sinus pauses/arrest, exit block, “inappropriate” sinus tachycardia and re-entrant arrhythmias, (Alonso et al., 2014; Jensen et al., 2014). It is largely a disease of the elderly and its incidence increases in an exponential manner with age (Mandel et al., 1999; Adan and Crown, 2003; Dobrzynski et al., 2007). Although human SND can be induced by a number of different pathophysiological mechanisms such as drug effects, autonomic imbalances, cardiomyopathy and electrophysiological alterations, SAN structural abnormalities have been commonly observed in these dysfunctions and are significantly associated with HF, SAN ischemia, and inflammatory conditions (Jordan et al., 1978; Adan and Crown, 2003; Sanders et al., 2004; Kottkamp, 2012). In 1907, Keith and Flack saw a “definite increase in the amount of fibrous tissue” present in the SAN from pathological hearts (Keith and Flack, 1907). Later, Hudson emphasized the association of extensive fibrotic lesions in the human SAN with established arrhythmia, especially AF, and suggested that temporary arrhythmias may be associated with minor fibrotic lesions in the SAN (Hudson, 1960). Thery et al. demonstrated a direct correlation between SAN fibrosis and the occurrence of tachy-brady syndrome (Thery et al., 1977). Tachy-brady syndrome, or tachy-brady arrhythmias, have been explained as “the heart rate alternating between too fast and too slow,” where termination of paroxysmal tachycardia “may be followed by long atrial pauses lasting several seconds, which can provoke another tachyarrhythmia paroxysm” (Figure 2A) (Moss and Davis, 1974; Lou et al., 2013, 2014). While the natural increase in SAN fibrosis observed with aging and heart size is not directly associated with SND, degenerative loss of SAN pacemaker cells and their replacement with fibrosis tissue is frequently evident upon pathologic examination of specimens from patients with SND (Birchfield et al., 1957; Davies and Pomerance, 1972; Thery et al., 1977; Hurle et al., 2006; Yeh et al., 2009).

### Excessive fibrosis leads to conduction blocks and reentry in the SAN pacemaker complex

Upregulated interstitial fibrosis may lead to disruption of the continuity of electrically coupled myocytes (Nguyen et al., 2014), which alters the delicate balance between depolarized cells (source) and the resting tissue ahead (sink), thereby disrupting SAN pacemakers' mutual entrainment (Jalife, 1984; Delmar et al., 1986; Michaels et al., 1987). As such, too much fibrosis slows activity of primary pacemaker clusters, as well as enhances beat-to-beat variability and atrial arrhythmias due to competition between multiple primary and subsidiary/latent pacemaker clusters within and outside the SAN pacemaker complex (Glukhov et al., 2013). Increased nodal fibrosis can cause slowed and decremental conduction within the SAN complex, especially in the SACPs (Figure 1D), leading to SAN exit block and long pauses, especially after tachycardia termination. Specifically, the atrial tachyarrhythmia or fast pacing depresses conduction/excitability in the structurally remodeled poorly coupled SACP (Figure 1D) which enhances source-sink mismatch and leads to exit block from the SAN after tachyarrhythmia termination, which can give rise to tachy-brady syndrome and cardiac arrest (Fedorov et al., 2010a; Glukhov et al., 2010, 2013; Hao et al., 2011; Swaminathan et al., 2011; Froese et al., 2012; Lou et al., 2014). Intramural optical mapping studies integrated with micro-structural analysis directly revealed that fibrosis could also lead to intranodal longitudinal and transverse conduction blocks and cause SAN micro- and macro-reentry (Glukhov et al., 2013; Lou et al., 2014) (Figures 2B–D). Interestingly, SAN macro-reentry (where signal travels from the one SACP to the atria and then back to the SAN through another SACP) will lead to tachycardia, but micro-reentry inside the SAN may lead to exit block in the SACP and severe bradycardia (Glukhov et al., 2013) (Figure 2). Histological analysis revealed that these arrhythmias required intranodal fibrotic strands not present in healthy hearts, indicating a critical role of intranodal fibrosis in creating structural substrates for SAN macro- and micro-reentry and atrial arrhythmias (Glukhov et al., 2013; Lou et al., 2014).

Importantly, disease-induced increases in interstitial fibrosis can abnormally insulate latent atrial pacemaker clusters thereby increasing their pacemaking properties (Boineau et al., 1988; Anderson et al., 2009; Atkinson et al., 2013; Dobrzynski et al., 2013). We recently demonstrated this novel mechanism in a mouse model of Calsequestrin 2 knockout, wherein increased pathological fibrosis indeed insulated latent atrial pacemaker clusters that competed with the SAN, causing heterogeneous conduction within the atria and subsequently AF and ventricular tachycardia (Glukhov et al., 2015). However, Verheule et al. found significant depression of atrial conduction and higher inducibility of AF in a mouse model of atria-specific TGF-β 1 overexpression but no evidence of SAN dysfunction, indicating upregulation of atrial fibrosis alone may not be sufficient to cause SAN dysfunction (Verheule et al., 2004).

It is not always clear whether fibrosis upregulation always precedes SAN dysfunction, or if fibrosis is generated as a response to SAN dysfunction. However, in SND animal models and recent clinical late-gadolinium MRI studies (Akoum and Marrouche, 2014) where structural remodeling in the SAN was studied, upregulated fibrosis in the SAN pacemaker complex was found (Sakabe et al., 2005; Hao et al., 2011; Herrmann et al., 2011; Swaminathan et al., 2011; Froese et al., 2012; Nakao et al., 2012; Glukhov et al., 2013; Wolf et al., 2013; Lou et al., 2014).

### Disease-induced fibroblast-myocyte coupling

Additionally, some studies have suggested that fibroblast-myocyte coupling (Kohl et al., 1994; Camelliti et al., 2004) can increase during pathological structural remodeling (e.g., MI and HF) (Kamkin et al., 2001, 2002; Vasquez et al., 2010); however, the existence of these electrical connections is still up for debate (Kohl and Gourdie, 2014). Depending on membrane potential, coupling and distribution, the fibroblast-pacemaker cell connections can increase or decrease the source-sink mismatch, thus accelerating or decelerating the pacemaking of the SAN (Fahrenbach et al., 2007). Increased coupling with interspersed fibroblasts may create abnormal cell-to-cell interactions as well as interrupt electrical coupling between SAN myocytes (decreased mutual entrainment) within the SAN complex, which has been shown in modeling (Oren and Clancy, 2010) and cell culture (Fahrenbach et al., 2007) studies. Furthermore, excessive extracellular matrix protein deposition by fibroblasts within the node can increase stiffness, which could impede the normal myogenic response of the SAN to changes in either stretch or arterial pressure, as mentioned above.

### Multiple mechanisms upregulate fibrosis in the SAN

Abnormal proliferation of fibroblasts and excessive secretion of ECM proteins have been shown to underlie cardiac fibrosis (Fan et al., 2012). The development of SAN arrhythmias have also been shown to occur in parallel with the loss of pacemaker cells (Hurle et al., 2006; Herrmann et al., 2011; Swaminathan et al., 2011; Wu and Anderson, 2014), leading to activated and reparative fibroblasts that replace dead cardiomyocytes and additional collagen deposition (Davies and Pomerance, 1972; Burstein and Nattel, 2008; Akoum et al., 2012). These deposits may form large, non-compliant areas of collagenous fibrosis devoid of cardiomyocytes, as in myocardial infarction(de Jong et al., 2011), or it may lead to increased intranodal fibrosis, as seen in HF (Figure 1D).

From the limited mechanistic studies on fibrosis within the SAN, there are multiple mechanisms believed to upregulate intranodal fibrosis. Myofibroblasts, the activated phenotype of fibroblasts, are known to underlie the excessive deposition of ECM proteins and are more mobile and contractile than fibroblasts (Davis and Molkentin, 2014). Overexpression of myocardial transforming growth factor β 1 (TGF-β 1) in mice has been shown to selectively increase fibrosis in the atria by causing a cellular transition from fibroblast to the activated myofibroblast phenotype (Nakajima et al., 2000; Vasquez et al., 2011). However, the presence of activated myofibroblasts is yet to be demonstrated in the SAN.

Another mechanism believed to be associated with increased fibrosis is the upregulation of Angiotensin II (Ang II). Circulating Ang II activates NADPH oxidase and increases oxidized calmodulin kinase II, leading to SAN cell apoptosis and fibrosis (Swaminathan et al., 2011). Ox-CaMKII-mediated loss of functional SAN cells contributes to SAN dysfunction and sudden cardiac death (Wu and Anderson, 2014). Recently, we demonstrated that fibrosis is increased within the SAN and could contribute to the slowed heart rate in a mouse model of catecholaminergic polymorphic ventricular tachycardia, a human arrhythmic disease (Glukhov et al., 2015). In this model of abnormal calcium (Ca^2+^) leak via the ryanodine receptors from the sarcoplasmic reticulum, increased fibrosis could either be due to upregulation of CaMKII secondary to altered Ca^2+^ signaling and/or SAN cell death leading to replacement with connective tissue.

Given the significant role that fibrotic remodeling plays in SND, anti-fibrotic approaches may present therapeutic options for this disease. For example, the ACE inhibitor Enalapril has been shown to reduce SAN fibrosis and SND in a four-week canine model of atrial tachycardia (Sakabe et al., 2005). Corticosteroids have also been shown to reduce TGF-β 1 and fibrosis in mouse lungs (Miller et al., 2006), and promising results in patients with complete AV block due to cardiac sarcoidosis have been observed (Selan et al., 2014). These results suggest that corticosteroids may prevent and/or restore fibrosis-induced SAN conduction impairment, but no study has specifically investigated if this approach is feasible.

## Future directions and conclusion

Even after one century of extensive research on the SAN, the lack of understanding of the human SAN complexity remains a critical barrier to the optimal treatment of heart rhythm disorders. Studies on explanted human SAN are necessary to determine its microstructure as well as structural and mechanistic role of fibrosis in normal as well as diseased hearts. Novel imaging techniques such as MRI or microCT (Akoum et al., 2012; Stephenson et al., 2012; Disertori et al., 2014) should be employed in addition to histological analyses to identify the intact structure as well as the contribution of fibrosis to SAN function and dysfunction.

Heterogeneous distribution of fibrosis within and around the SAN pacemaker complex plays a crucial role in proper SAN function by providing (1) structural and functional integrity/stability of the SAN (2) electrical insulation of SAN myocyte clusters and the entire SAN complex, apart from SACPs, preventing depression of pacemaker automaticity from the hyperpolarizing effect of the surrounding atria (3) mechanical protection from pathophysiological heart rate changes due to stretch and pressure from the contractile force of the atria.

The protective role that fibrosis plays in healthy hearts is necessary for proper SAN function, but this role becomes pathophysiological when fibrosis is upregulated in cardiac diseases, leading to (1) slowed SAN rhythm due to fibrosis replacement of pacemaker cells (2) beat-to-beat variability and slowed intranodal conduction due to decreased electrical coupling between SAN myocytes and pacemaker cell clusters (3) long pauses due to source–sink mismatch causing exit block in the SACPs (4) SAN micro- and macro-reentry due to fibrosis-induced conduction dissociation in the SAN. All of these pathological conditions can lead to arrhythmias such as tachy-brady syndrome and AF. By continuing to expand our knowledge of the complex structure that makes up the SAN and exploring the multiple mechanisms involved in fibrosis upregulation, we will come closer to providing new and less invasive treatment options for a growing population of aging patients suffering from SND.

## Funding

This work was supported by National Institute of Health HL115580 (to Vadim V. Fedorov), National Scientist Development Grant from the American Heart Association (to Anuradha Kalyanasundaram) and by the International Mobility Fund from Royal Society of New Zealand (to Jichao Zhao and Vadim V. Fedorov).

### Conflict of interest statement

The authors declare that the research was conducted in the absence of any commercial or financial relationships that could be construed as a potential conflict of interest.
